# Early Versus Delayed Laparoscopic Cholecystectomy for Acute Calculous Cholecystitis: A Systematic Review

**DOI:** 10.7759/cureus.114352

**Published:** 2026-08-11

**Authors:** Abeer A Hakam‎i, Maryam A Sultan, Hajar A Hak‎ami, Amjad A Durayb, Lujain A AlYousfi, Fatimah E Ageeli, Amani B Aburasain, Ibrahim A Homadi, Sultan O Gohal

**Affiliations:** 1 General Surgery, Jazan Health Cluster, Jazan, SAU

**Keywords:** acute calculous cholecystitis, conversion to open surgery, delayed cholecystectomy, early cholecystectomy, hospital stay, laparoscopic cholecystectomy, postoperative complications, randomized controlled trial, systematic review, timing of surgery

## Abstract

Acute calculous cholecystitis is a common emergency surgical condition, and the optimal timing of laparoscopic cholecystectomy (LC) remains controversial. Previous meta-analyses have not consistently distinguished evidence from randomized controlled trials (RCTs) and observational studies or separated postoperative complications from composite strategy-level morbidity. We conducted a systematic review and meta-analysis in accordance with the Preferred Reporting Items for Systematic Reviews and Meta-Analyses (PRISMA) 2020 guidelines by searching PubMed, the Cochrane Library, Scopus, and Web of Science from inception through June 2026. Dichotomous outcomes from RCTs were pooled using the DerSimonian-Laird random-effects model, while observational studies were synthesized narratively. Risk of bias was assessed using the revised Cochrane Risk of Bias 2 (RoB 2) tool and the Risk of Bias In Non-randomized Studies of Interventions (ROBINS-I) tool. Seventeen studies met the inclusion criteria (11 RCTs and six observational studies). Meta-analysis of RCTs demonstrated that early LC did not significantly increase conversion to open surgery (odds ratio (OR) 0.90, 95% confidence interval (CI) 0.58-1.38; I² = 0%), bile leak (OR 1.80, 95% CI 0.69-4.66), bile duct injury (OR 0.61, 95% CI 0.15-2.48), mortality (OR 0.94, 95% CI 0.21-4.17), or postoperative complications (OR 0.73, 95% CI 0.38-1.40). In contrast, composite strategy morbidity, which incorporated waiting-period events, was significantly lower with early LC (OR 0.26, 95% CI 0.17-0.38; p < 0.001; I² = 0%). Total hospital stay was consistently reduced by approximately three to six days across all 11 RCTs. Observational studies supported same-admission surgery but were uniformly at serious risk of confounding. Overall, early LC appears surgically safe and substantially reduces composite strategy morbidity and hospital stays without increasing operative risk. The traditional 72-hour symptom threshold should not be considered an absolute contraindication to surgery. Further high-quality evidence is needed for patients presenting beyond 72 hours, older adults, and severity-stratified subgroups.

## Introduction and background

Acute calculous cholecystitis is one of the most common emergency surgical conditions worldwide and represents a major cause of emergency general surgical admissions [1-3]. It results from cystic duct obstruction by gallstones, leading to gallbladder inflammation that may progress to ischemia, gangrene, or perforation. Diagnosis is based on clinical features, laboratory findings, and imaging. Initial management includes analgesia, intravenous fluid resuscitation, and antibiotics, followed by definitive treatment with surgery [4-9].

Laparoscopic cholecystectomy (LC) is the standard treatment for patients who are suitable surgical candidates [1,5,8]. Current guidelines recommend early LC, ideally within 72 hours of symptom onset. Although management has shifted from interval cholecystectomy after initial conservative treatment toward early surgery, delayed intervention is still practiced in many institutions because of resource limitations, surgeon availability, and other logistical factors [5-10].

The principal controversy is whether performing LC during the acute inflammatory phase increases operative difficulty or the risk of complications, such as conversion to open surgery or bile duct injury, compared with delayed surgery. Randomized controlled trials (RCTs) have generally shown that early LC is safe and reduces total hospital stay without increasing the risk of postoperative complications [1-11]. These findings have been supported by previous systematic reviews and meta-analyses.

Despite these findings, several important issues remain unresolved. Definitions of "early" surgery vary across studies, limiting direct comparisons. Additionally, many reviews have combined postoperative complications with composite strategy-level morbidity, which also includes treatment failures, recurrent cholecystitis, unplanned readmissions, and emergency surgery during the waiting period for delayed intervention [3-9]. This approach may underestimate the overall clinical burden associated with delayed surgery. Furthermore, the widely used 72-hour symptom threshold is supported by limited randomized evidence, while elderly patients and those with substantial comorbidities remain underrepresented in clinical trials. Observational studies evaluating the timing of surgery during the same hospital admission provide complementary evidence but should be interpreted separately from randomized data because of their greater susceptibility to confounding [12-17].

This systematic review and meta-analysis aimed to evaluate the impact of LC timing on surgical and clinical outcomes in acute calculous cholecystitis by using RCTs as the primary quantitative evidence base and observational studies as supportive narrative evidence. Particular emphasis was placed on distinguishing postoperative complications from composite strategy-level morbidity and assessing the evidence surrounding the 72-hour threshold for early surgery.

## Review

Methods

Study Design

This systematic review and meta-analysis was conducted and reported in accordance with the Preferred Reporting Items for Systematic Reviews and Meta-Analyses (PRISMA) 2020 guidelines and followed the recommendations of the Cochrane Handbook for Systematic Reviews of Interventions [18].

Eligibility Criteria

Two reviewers independently screened titles, abstracts, and full-text articles for eligibility according to the predefined inclusion and exclusion criteria (Table 1). Disagreements were resolved by consensus or consultation with a third reviewer. Data were extracted using a standardized form that captured study characteristics, patient populations, intervention and comparator definitions, and outcome data.

**Table 1:** Inclusion and exclusion criteria for study selection Eligibility criteria were applied during the study selection process for the systematic review. Studies were included if they evaluated adult patients with acute calculous cholecystitis and compared early or same-admission LC with delayed LC. Studies were excluded if they did not meet the predefined population, intervention, comparator, study design, or outcome requirements. RCT: randomized controlled trial, LC: laparoscopic cholecystectomy

Criteria type	Criterion
Inclusion	Adult patients (≥18 years) diagnosed with acute calculous cholecystitis
	Studies comparing early or same-admission LC with delayed LC
	Early LC performed within 24, 48, or 72 hours of symptom onset or admission, or during the index admission according to the study definition
	Delayed LC performed after initial conservative management, typically 6-12 weeks later
	RCTs and comparative observational studies
	Studies reporting extractable data for at least one predefined clinical outcome
Exclusion	Pediatric populations
	Elective LC performed without acute gallbladder inflammation
	Non-comparative studies, case reports, and case series
	Reviews, editorials, letters, conference abstracts, and other non-original publications
	Non-English publications
	Studies without sufficient outcome data for extraction or quantitative/narrative synthesis

Search Strategy

PubMed/MEDLINE, the Cochrane Library, Scopus, and Web of Science were searched from database inception to 15 June 2026. The database-specific search strategies are summarized in Table 2. Search terms combined concepts related to acute calculous cholecystitis, LC, and surgical timing (including early, delayed, same-admission, and interval surgery). No restrictions on publication date or study design were applied.

**Table 2:** Electronic database search strategies used for literature identification Database-specific search strategies used to identify studies comparing early versus delayed LC for acute calculous cholecystitis. Searches were performed in PubMed/MEDLINE, the Cochrane Library (CENTRAL), Scopus, and Web of Science from database inception to 15 June 2026. Search terms combined controlled vocabulary and free-text terms related to acute cholecystitis, LC, and timing of surgical intervention. No restrictions on publication date or study design were applied. MeSH: Medical Subject Headings, tiab: title/abstract field, TS: topic search field, TITLE-ABS-KEY: title, abstract, and keywords field, LC: laparoscopic cholecystectomy

Database	Search strategy
PubMed/MEDLINE	("cholecystitis, acute"[MeSH] OR "acute cholecystitis"[tiab] OR "acute calculous cholecystitis"[tiab]) AND ("cholecystectomy, laparoscopic"[MeSH] OR "laparoscopic cholecystectomy"[tiab] OR "cholecystectomy"[tiab]) AND ("early"[tiab] OR "delayed"[tiab] OR "immediate"[tiab] OR "interval"[tiab] OR "same admission"[tiab] OR "index admission"[tiab] OR "24 hours"[tiab] OR "48 hours"[tiab] OR "72 hours"[tiab] OR "timing"[tiab])
Cochrane Library (CENTRAL)	#1 "acute cholecystitis" in Title Abstract Keyword #2 "laparoscopic cholecystectomy" OR "cholecystectomy" in Title Abstract Keyword #3 "early" OR "delayed" OR "immediate" OR "interval" OR "timing" OR "same admission" OR "24 hours" OR "48 hours" OR "72 hours" in Title Abstract Keyword #4 #1 AND #2 AND #3
Scopus	TITLE-ABS-KEY("acute cholecystitis") AND TITLE-ABS-KEY("laparoscopic cholecystectomy" OR "cholecystectomy") AND TITLE-ABS-KEY("early" OR "delayed" OR "immediate" OR "interval" OR "timing" OR "same admission" OR "index admission" OR "24 hours" OR "48 hours" OR "72 hours")
Web of Science	TS=("acute cholecystitis") AND TS=("laparoscopic cholecystectomy" OR "cholecystectomy") AND TS=("early" OR "delayed" OR "immediate" OR "interval" OR "timing" OR "same admission" OR "index admission" OR "24 hours" OR "48 hours" OR "72 hours")

Study Selection and Data Extraction

Two reviewers independently screened titles, abstracts, and full-text articles for eligibility. Disagreements were resolved by consensus or consultation with a third reviewer. Data were extracted using a standardized form that captured study characteristics, patient populations, intervention and comparator definitions, and outcome data. When reporting inconsistencies or incomplete data were identified, they were documented and considered during sensitivity analyses.

Outcomes

The primary outcomes were conversion to open surgery, bile leak, bile duct injury, mortality, postoperative complications, and composite strategy morbidity. Composite strategy morbidity included treatment failure, recurrent acute cholecystitis, unplanned readmission or emergency consultation, emergency surgery before planned delayed cholecystectomy, and postoperative complications. Total hospital stay was summarized narratively because insufficient data were available for quantitative pooling.

Risk of Bias Assessment

Risk of bias in RCTs was assessed using the revised Cochrane Risk of Bias 2 (RoB 2) tool [19]. Observational studies were evaluated using the Risk of Bias in Non-randomized Studies of Interventions (ROBINS-I) tool [20].

Data Synthesis and Statistical Analysis

RCTs and observational studies were analyzed separately. Only RCTs were included in the quantitative meta-analysis, whereas observational studies were synthesized narratively. Pooled odds ratios (ORs) with 95% confidence intervals (CIs) were calculated using the DerSimonian-Laird random-effects model. A continuity correction of 0.5 was applied to zero-event studies. Statistical heterogeneity was assessed using the I² statistic. Prespecified sensitivity analyses evaluated the impact of studies with uncertain denominators, risk of bias, and analysis populations. Statistical analyses and forest plots were generated using Python.

Results

Study Selection and Characteristics

**Figure 1:**
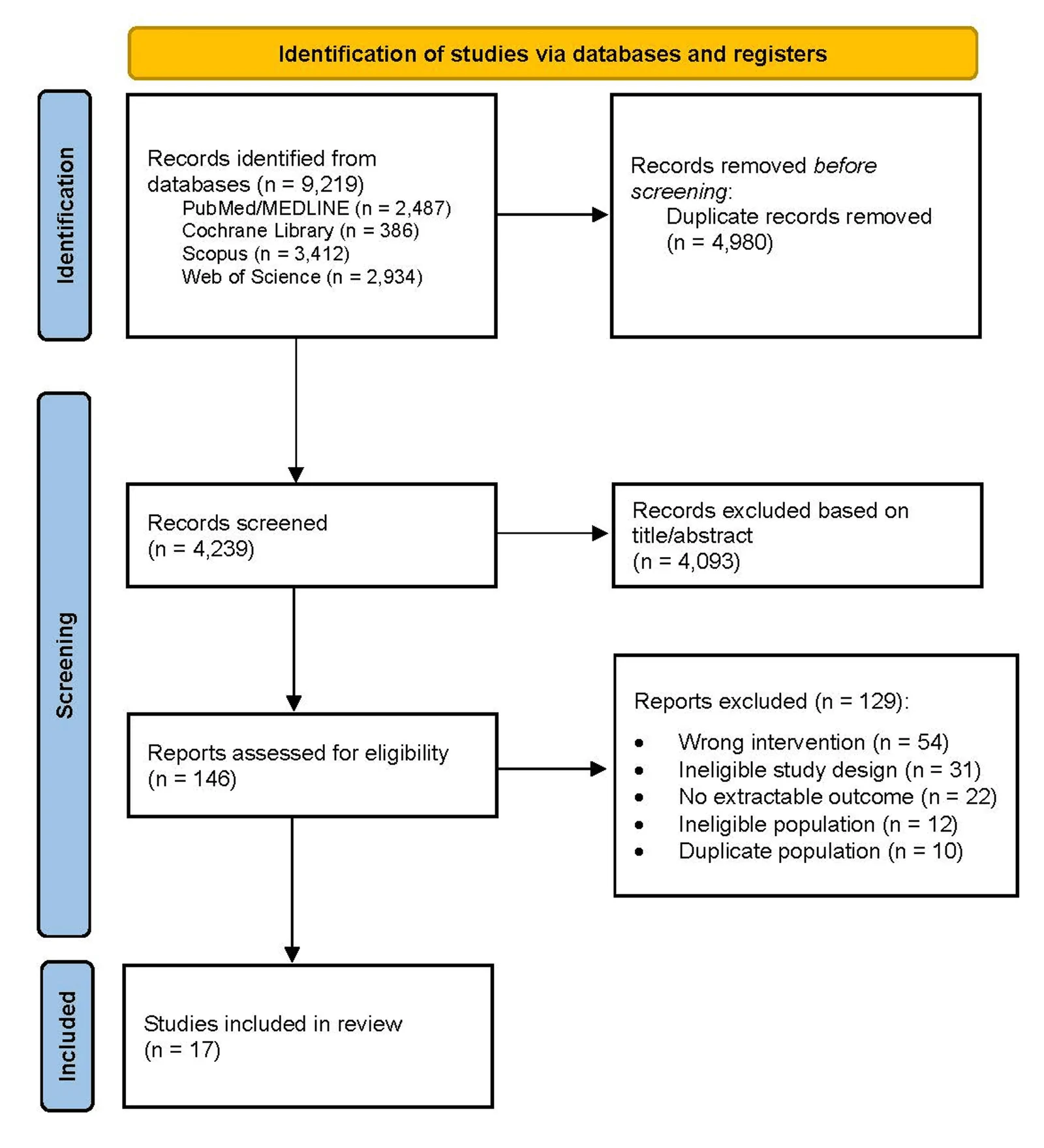
PRISMA flow diagram depicting the study selection process for the systematic review PRISMA flow diagram detailing the study selection process for this systematic review. After identifying records through database searching and other sources, duplicates were removed, and the remaining records were screened. Full-text articles were assessed for eligibility, with exclusions documented along with reasons. Studies meeting the inclusion criteria were included in the final synthesis. PRISMA: Preferred Reporting Items for Systematic Reviews and Meta-Analyses

Figure 1 summarizes the study selection process according to the PRISMA 2020 guidelines. The search identified 9,219 records. After removal of duplicates, 4,239 records were screened, and 146 full-text articles were assessed for eligibility. Seventeen studies were included. Eleven were RCTs comparing early versus delayed LC and represented the primary quantitative evidence base [1-11]. Six observational studies evaluating the timing of surgery during the index admission were included for narrative synthesis only [12-17]. Randomized and observational studies were analyzed separately (Tables 3-4).

**Table 3:** Characteristics of included RCTs comparing early and delayed LC Characteristics of the included RCTs evaluating early versus delayed LC for acute calculous cholecystitis. Data are presented by study identifier, country, study design, analyzed sample size in the early and delayed groups, and definitions of early and delayed LC. A conservative treatment strategy refers to initial non-operative management followed by elective LC after the specified interval. †Roulin et al. enrolled patients with symptom duration >72 hours. n: number of analyzed patients, RCT: randomized controlled trial, LC: laparoscopic cholecystectomy

Study ID	Country	Design	n (early/delayed)	Early definition	Delayed definition
Gul et al., 2013 [1]	India	Prospective RCT; single center	30/30	Within 72 h of admission	Conservative treatment → elective LC 6-12 weeks later
Gutt et al., 2013 [2]	Germany/Slovenia	Multicenter prospective RCT; 35 centers	304/314	Within 24 h of admission	Moxifloxacin treatment → elective LC 7-45 days later
Johansson et al., 2003 [3]	Sweden	Prospective RCT; single center	74/69	Within 48 h of randomization; ≤7 days from onset	Conservative treatment → elective LC 6-8 weeks later
Kolla et al., 2004 [4]	India	Prospective RCT; single center	20/20	Within 24 h of randomization	Conservative treatment → elective LC 6-12 weeks later
Lai et al., 1998 [5]	China	Prospective RCT; single center	53/38	Within 24 h of randomization	Conservative treatment → elective LC 6-8 weeks later
Yadav et al., 2009 [6]	Nepal	Prospective RCT; single center	25/25	As soon as possible (mean ~109 h from onset)	Conservative treatment → elective LC 6-8 weeks later
Lo et al., 1998 [7]	China	Prospective RCT; single center	45/41	Within 72 h of admission (median 31 h)	Conservative treatment → elective LC 8-12 weeks later
Özkardeş et al., 2014 [8]	Turkey	Prospective RCT; single center	30/30	Within 24 h of admission	Conservative treatment → elective LC 6-8 weeks later
Rajcok et al., 2016 [9]	Slovakia	Prospective RCT; single center	31/31	Within 72 h of symptom onset	Conservative treatment → elective LC 6-8 weeks later
Roulin et al., 2016 [10]	Switzerland	Prospective RCT; single center	41/41	As soon as possible after admission	Antibiotics → elective LC ≥6 weeks later
Saber et al., 2014 [11]	Egypt	Prospective RCT; two centers	60/60	Within 72 h of symptom onset	Conservative treatment → elective LC 6-8 weeks later

**Table 4:** Characteristics of included observational studies evaluating timing of LC Characteristics of observational studies assessing the association between timing of LC and clinical outcomes in acute calculous cholecystitis. Data include study setting, design, sample size, timing exposure definitions, and reported outcomes. Timing categories represent the exposure definitions used in each individual study. n: number of analyzed patients, LC: laparoscopic cholecystectomy, SALTS: Swiss Association of Laparoscopic and Thoracoscopic Surgery, ACS: American College of Surgeons, NSQIP: National Surgical Quality Improvement Program, NIS: Nationwide Inpatient Sample, LOS: length of stay, SSI: surgical site infection, UTI: urinary tract infection

Study ID	Country/source	Design	n	Exposure/timing	Key outcomes
Banz et al., 2011 [12]	Switzerland; SALTS database	Prospective cohort; population-based	4113	Day of admission to surgery: d0, d1, d2, d3, d4-5, d≥6	Conversion, complications, reoperation, LOS, mortality
Brooks et al., 2013 [13]	USA; ACS-NSQIP 2005-2010	Retrospective database cohort	14068	Preoperative LOS: 0, 1, 2, 3, 4-7 days	30-day morbidity, mortality, LOS, operative time
Zafar et al., 2015 [14]	USA; NIS 2005-2009	Retrospective database cohort	95523	Days 0-1, 2-5, 6-10 from presentation	Mortality, SSI, pneumonia, UTI, LOS, cost
Degrate et al., 2013 [15]	Italy; single center	Retrospective cohort	316	Same-admission LC vs delayed LC (≥4 weeks); subgroup: ≤72 h vs >72 h from symptom onset	Conversion, complications, bile leak, LOS
Fugazzola et al., 2023 [16]	Italy/multicenter; S.P.Ri.M.A.C.C.	Post-hoc analysis of prospective cohort	1117	0-3 days, 4-7 days, 8-10 days from symptom onset	Intraoperative complications, conversion, major complications, mortality, LOS
Haltmeier et al., 2015 [17]	USA; ACS-NSQIP 2005–2010	Retrospective database cohort; age >65 years	4011	Day 0 (early) vs day >0 (delayed) same-admission LC	Mortality, complications, LOS, conversion

Risk of Bias Assessment

Risk of bias was assessed using the revised RoB 2 and ROBINS-I tools. Among the 11 RCTs, three studies were judged to have some concerns. Among the 11 RCTs, six were judged to have some concerns, and five were considered at high risk of bias, mainly due to unclear randomization methods, post-randomization exclusions, and reporting limitations. All observational studies were considered at serious risk of bias, primarily because treatment timing was influenced by baseline differences in patient characteristics, disease severity, and comorbidity (Tables 5-6).

**Table 5:** Domain-level risk of bias assessment of included RCTs using the RoB 2 tool Risk of bias assessment for included RCTs using the revised RoB 2 tool. Domains assessed were D1, bias arising from the randomization process; D2, bias due to deviations from intended interventions; D3, bias due to missing outcome data; D4, bias in measurement of the outcome; and D5, bias in selection of the reported result. Overall risk of bias was classified as low risk, some concerns, or high risk according to the RoB 2 criteria. RCT: randomized controlled trial, RoB 2: Cochrane Risk of Bias 2

Study ID	D1: randomization process	D2: deviations from intended interventions	D3: missing outcome data	D4: measurement of outcome	D5: selection of reported result	Overall risk of bias
Gul et al., 2013 [1]	High risk	Some concerns	Some concerns	Some concerns	High risk	High risk
Gutt et al., 2013 [2]	Some concerns	Some concerns	Some concerns	Some concerns	Low	Some concerns
Johansson et al., 2003 [3]	Some concerns	Some concerns	Some concerns	Low	Some concerns	Some concerns
Kolla et al., 2004 [4]	Some concerns	Some concerns	Some concerns	Some concerns	Some concerns	Some concerns
Lai et al., 1998 [5]	Some concerns	Some concerns	Some concerns	Some concerns	Some concerns	Some concerns
Yadav et al., 2009 [6]	High risk	Some concerns	High risk	High risk	High risk	High risk
Lo et al., 1998 [7]	Some concerns	Some concerns	Some concerns	Some concerns	Some concerns	Some concerns
Özkardeş et al., 2014 [8]	High risk	Some concerns	Some concerns	Some concerns	High risk	High risk
Rajcok et al., 2016 [9]	High risk	Some concerns	High risk	High risk	High risk	High risk
Roulin et al., 2016 [10]	Some concerns	Some concerns	Some concerns	Some concerns	Some concerns	Some concerns
Saber et al., 2014 [11]	High risk	Some concerns	Some concerns	Some concerns	High risk	High risk

**Table 6:** Domain-level risk of bias assessment of included observational studies using the ROBINS-I tool Risk of bias assessment for included observational studies using the ROBINS-I tool. Domains assessed included confounding, selection bias, classification of interventions, deviations from intended interventions, missing data, measurement of outcomes, and reporting bias. Overall risk of bias was classified according to the ROBINS-I criteria. ROBINS-I: Risk of Bias in Non-randomized Studies of Interventions

Study ID	Confounding	Selection bias	Classification of interventions	Deviations from intended interventions	Missing data	Measurement of outcomes	Reporting bias	Overall risk of bias
Banz et al., 2011 [12]	Serious	Moderate	Moderate	Moderate	Low	Moderate	Moderate	Serious
Brooks et al., 2013 [13]	Serious	Moderate	Moderate	Moderate	Moderate	Moderate	Moderate	Serious
Zafar et al., 2015 [14]	Serious	Moderate	Moderate	Moderate	Moderate	Serious	Moderate	Serious
Degrate et al., 2013 [15]	Serious	Moderate	Moderate	Moderate	Moderate	Moderate	Moderate	Serious
Fugazzola et al., 2023 [16]	Serious	Moderate	Moderate	Moderate	Moderate	Moderate	Moderate	Serious
Haltmeier et al., 2015 [17]	Serious	Moderate	Moderate	Moderate	Moderate	Moderate	Moderate	Serious

Summary of Outcomes

Six prespecified outcomes were analyzed using pooled ORs with 95% CIs using a random-effects model. Statistical heterogeneity was minimal across outcomes. Early LC did not significantly increase conversion to open surgery (nine RCTs; n = 734; OR 0.90, 95% CI 0.58-1.38; p = 0.622; I² = 0%) [1-11]. Sensitivity analyses including studies with uncertain denominators and analyses restricted to lower-risk trials showed similar results (Table 7).

**Table 7:** Summary of pooled random-effects meta-analysis results Summary of pooled random-effects meta-analysis estimates comparing early versus delayed LC for acute calculous cholecystitis. Outcomes are presented as pooled ORs with corresponding 95% CIs, p-values, heterogeneity statistics, and analysis type. Main analyses represent prespecified primary outcome analyses, whereas sensitivity analyses represent additional analyses assessing robustness of findings. Postoperative complications (restricted) included only studies with reliable patient-level denominators. Composite strategy morbidity included treatment failure, recurrent acute cholecystitis, unplanned readmission or emergency consultation, emergency surgery before planned delayed LC, and postoperative complications. A continuity correction of 0.5 was applied to zero-event cells. Bile duct injury and mortality estimates should be interpreted cautiously because of the limited number of events. k: number of contributing studies, n: number of analyzed patients, OR: odds ratio, CI: confidence interval, I²: measure of statistical heterogeneity, τ²: between-study variance estimated using the DerSimonian-Laird method, LC: laparoscopic cholecystectomy

Outcome	k	n	Pooled OR	95% CI	p-value	I²	τ²	Analysis type
Conversion to open surgery	9	734	0.90	0.58-1.38	0.622	0	0	Main
Bile leak	8	1225	1.80	0.69-4.66	0.228	0	0	Main
Bile duct injury	6	502	0.61	0.15-2.48	0.49	0	0	Main (caution)
Mortality	6	1098	0.94	0.21-4.17	0.936	0	0	Main (insufficient events)
Postoperative complications (restricted)	4	299	0.73	0.38-1.40	0.343	0	0	Main
Composite strategy morbidity	2	704	0.26	0.17-0.38	<0.001	0	0	Main
Conversion + Özkardeş	10	794	0.94	0.62-1.44	0.79	0	0	Sensitivity
Conversion + Gutt	10	1040	0.89	0.64-1.25	0.509	0	0	Sensitivity
Conversion - low/some risk-of-bias only	5	449	0.90	0.55-1.46	0.674	0	0	Sensitivity
Bile leak + Özkardeş	9	1285	1.88	0.75-4.68	0.177	0	0	Sensitivity
Postoperative complications + Rajcok	5	361	0.61	0.34-1.10	0.1	0	0	Sensitivity
Postoperative complications + Özkardeş	5	359	1.05	0.41-2.68	0.915	0.484	0.236	Sensitivity
Strategy morbidity (per-protocol)	2	636	0.27	0.18-0.41	<0.001	0	0	Sensitivity

Bile leak was reported in eight RCTs (n = 1,225) and was not significantly different between early and delayed LC (OR 1.80, 95% CI 0.69-4.66; p = 0.228; I² = 0%) [1-11]. Bile duct injury was reported in six RCTs (n = 502), with very few events and substantial uncertainty (OR 0.61, 95% CI 0.15-2.48; p = 0.490; I² = 0%) [1-11]. Mortality was rare, with only two events across six RCTs (n = 1,098), and no significant difference was observed between groups (OR 0.94, 95% CI 0.21-4.17; p = 0.936; I² = 0%) [1-11].

Postoperative complications were evaluated in four RCTs with reliable patient-level denominators (n = 299). Early LC showed a nonsignificant reduction in postoperative complications (OR 0.73, 95% CI 0.38-1.40; p = 0.343; I² = 0%) [1-11]. Sensitivity analyses produced similar estimates.

Composite strategy morbidity, which incorporated treatment failure, recurrent cholecystitis, unplanned readmission or emergency consultation, emergency surgery before planned delayed LC, and postoperative complications, was reported in two RCTs [2,10]. Early LC significantly reduced composite morbidity compared with delayed surgery (OR 0.26, 95% CI 0.17-0.38; p < 0.001; I² = 0%). This effect remained consistent in per-protocol sensitivity analysis (OR 0.27, 95% CI 0.18-0.41; p < 0.001).

Hospital Stay

A quantitative meta-analysis of hospital stay was not performed because insufficient studies provided compatible mean and standard deviation data. However, all 11 RCTs consistently demonstrated shorter hospitalization with early LC, with reported reductions of approximately three to six days compared with delayed surgery [1-11].

Observational Evidence

Observational studies supported early same-admission LC but were interpreted cautiously because of substantial confounding. Population-based studies suggested that longer delays during the index admission were associated with increased conversion rates, postoperative morbidity, and healthcare costs [12-14]. However, these findings cannot establish causality because surgical timing was not randomized.

Studies evaluating the 72-hour symptom threshold found no significant increase in conversion, postoperative complications, or bile leak among patients undergoing surgery beyond 72 hours during the same admission [15]. Prospective multicenter data suggested that operative difficulty increased with longer delays, particularly beyond 7 days, while major complications and mortality remained similar across timing groups [16]. The only RCT specifically including patients presenting beyond 72 hours demonstrated that early LC did not increase operative complications and reduced composite strategy morbidity and hospital stay [10].

In elderly and higher-risk patients, early LC was associated with a shorter hospital stay without a significant increase in mortality or postoperative complications. However, the observational design and residual confounding limit causal interpretation [17].

Limitations

This review has several limitations. Most RCTs were at high risk of bias, with inconsistent definitions of early and delayed surgery across studies. Rare events such as bile duct injury and mortality limited the precision of effect estimates. Hospital stay data could not be quantitatively pooled because of inconsistent reporting. Observational studies were subject to substantial confounding and potential misclassification. Additionally, the absence of prospective protocol registration may have introduced methodological concerns.

## Conclusions

Early LC is an effective management approach for appropriately selected patients with acute cholecystitis. It offers important clinical benefits by reducing overall treatment burden and hospital resource utilization while maintaining an acceptable safety profile. The decision regarding timing of surgery should consider individual patient factors, disease severity, and local surgical expertise rather than relying on a fixed time threshold alone. Further research is needed to better define optimal management strategies for higher-risk populations and specific clinical subgroups.
